# Supplementary material for: Use of seat belts among public transport drivers in Tacna, Peru: Prevalence and risk factors
Source: PLoS One. 2021 May 18;16(5):e0251794. doi: 10.1371/journal.pone.0251794 (PMC8130960; doi:10.1371/journal.pone.0251794)
Supplement: S1 File — (PDF) [file pone.0251794.s001.pdf]

APPENDIX 1: Questionnaire

QUESTIONNAIRE

Use of seat belts in public transport drivers in the city of Tacna

Dear driver: Your participation in this research is voluntary, and its objective is to observe the characteristics of the use of safety devices in public transport drivers. The data provided is confidential and anonymous. The results will be published as aggregated data in a scientific journal.

Thank you very much for your help

Survey N °: ___________ Survey date: ___________ Survey time: _________

GENERAL CHARACTERISTICS

1. Sex: a) female b) male

2. Age: _____ years of age

3. Level of instruction: a) None b) Incomplete primary c) Complete primary d) Incomplete secondary d) Complete secondary e) Superior

4. Marital status: a) Single b) Cohabiting c) Married d) Divorced

5. Do you have children? a) No b) Yes

> If you have children: How many children do you have? _____

6. Type of service: a) Taxi b) Buses

> If it is a taxi: Does it belong to a Radiotaxi company? a) No b) Yes

> If it is a bus: Are you a collector driver? a) No b) Yes

7. What kind of driving license you have? a) AI b) AII-a c) AII-b d) AIII-a e) AIII-b f) AIII-c

8. How many hours have you been working today? ______

9. How many hours do you work a day? ______

10. How many years have you been working as a driver? ______

11. Have you received any road safety courses in the last 12 months? a) No b) Yes

12. In the last 6 months, have you received any traffic ballots? a) No b) Yes

TRAFFIC ACCIDENTS

13. Have you ever had a traffic fine as a driver? a) No b) Yes

> If you answered yes:

How many traffic accidents have you had as a driver? __________

Have you had a traffic accident in the last 12 months? a) No b) Yes

How long ago was the last accident? _____________ Months years

Did you go to a health facility for a traffic accident? No b) Yes

USE OF THE MOBILE PHONE

14. Have you ever used your cell phone while driving? a) No b) Yes

15. Did you use your cell phone during the survey? a) No b) Yes

USE OF THE SEAT BELT

16. The driver do you use a seat belt ?: a) No b) Yes

> Type of seat belt anchorage: a) 2 points b) 3 points

> If not using seat belts: Why not wear a seat belt? ________________________

17. In the seat of the co-driver's seat belt is there ?: a) No b) Yes

> Type of seat belt anchorage: a) 2 point b) 3 points
