# Supplementary material for: Use of seat belts among public transport drivers in Tacna, Peru: Prevalence and risk factors
Source: PLoS One. 2021 May 18;16(5):e0251794. doi: 10.1371/journal.pone.0251794 (PMC8130960; doi:10.1371/journal.pone.0251794)
Supplement: S2 File — (PDF) [file pone.0251794.s002.pdf]

Uso de cinturón de seguridad en conductores de transporte público en la ciudad de Tacna

Estimado conductor: Su participación en esta investigación es voluntaria, y tiene como objetivo observar las características del uso de dispositivos de seguridad en conductores de transporte público. Los datos proporcionados son confidenciales y anónimos. Los resultados serán publicados como datos agregados en una revista científica.

Muchas gracias por su colaboración

N° Encuesta: _______ Fecha encuesta:____________ Hora encuesta:_______

CARACTERÍSTICAS GENERALES

1. **Sexo:** a) femenino b) masculino
2. **Edad:** _____ años cumplidos
3. **Grado de instrucción:** a) Ninguna b) Primaria incompleta c) Primaria completa d) Secundaria incompleta d) Secundaria completa e) Superior
4. **Estado civil:** a) Soltero b) Conviviente c) Casado d) Divorciado
5. **¿Tiene hijos?** a) No b) Si
   >Si tiene hijos: ¿Cuántos hijos tiene? _____
6. **Tipo de servicio:** a) Taxi b) Buses
   > Si es taxi: ¿Pertenece a una empresa de Radiotaxi? a) No b) Si
   > Si es bus: ¿Es chofer cobrador? a) No b) Si
7. **¿Qué tipo de brevete tiene?** a) AI b) AII-a c) AII-b d) AIII-a e) AIII-b f)AIII-c
8. **¿Cuántas horas lleva trabajando hoy?** ______
9. **¿Cuántas horas trabaja al día?** ______
10. **¿Cuántos años lleva trabajando como conductor?** ______
11. **¿Ha recibido algún curso de seguridad vial en los últimos 12 meses?**
    a) No b) Si
12. **En los últimos 6 meses ¿ha recibido alguna papeleta de tránsito?** a) No b) Si

ACCIDENTES DE TRÁNSITO

13. **¿Alguna vez ha tenido un accidente de tránsito como conductor?** a) No b) Si
    > Si respondió si:
    ¿Cuántos accidentes de tránsito como conductor ha tenido? __________
    ¿Ha tenido algún accidente de tránsito en los últimos 12 meses? a) No b) Si
    ¿Hace cuánto tiempo fue el último accidente? _____________ meses / años
    ¿Acudió a un establecimiento de salud por algún accidente de tránsito?
    a) No b) Si

USO DEL DISPOSITIVO MÓVIL

14. **¿Alguna vez ha utilizado el celular mientras manejaba?** a) No b) Si
15. **¿Utilizó el celular durante la encuesta?** a) No b) Si

USO DEL CINTURÓN DE SEGURIDAD

16. **El conductor ¿usa el cinturón de seguridad?:** a) No b) Si
    > Tipo de anclaje del cinturón de seguridad: a) 2 puntos b) 3 puntos
    > Si no usa el cinturón de seguridad: ¿Por qué no usa el cinturón de seguridad?
    ____
17. **En el asiento del copiloto ¿hay cinturón de seguridad?:** a) No b) Si
    > Tipo de anclaje del cinturón de seguridad: a) 2 puntos b) 3 puntos
