# Supplementary material for: Use of seat belts among public transport drivers in Tacna, Peru: Prevalence and risk factors
Source: PLoS One. 2021 May 18;16(5):e0251794. doi: 10.1371/journal.pone.0251794 (PMC8130960; doi:10.1371/journal.pone.0251794)
Supplement: Attachment — Submitted filename: 210329_Response to the Reviewers.docx [file pone.0251794.s003.docx]

**Response to the Reviewers**

1. **Comments of the reviewers:** In the ethics statement in the Methods section and online submission information, please specify the type of informed consent that was obtained from the participants (for instance, written or verbal, and if verbal, how it was documented and witnessed).

**Comments of the authors:** The authors of the study got a verbal consent from the participants. This verbal consent was obtained before the application of the study. It was witnessed by the pollsters, but it was not documented (to reduce rejection rate). Outcome evaluation (use the cellphone and seat belt use) was performed since the pollsters got into the vehicle (to avoid information bias), being one of the first reports of the use of seat belt in public transport drivers assessed from inside the vehicle

2. **Comments of the reviewers:** Please include additional information regarding the survey or questionnaire used in the study and ensure that you have provided sufficient details that others could replicate the analyses. For instance, if you developed a questionnaire as part of this study and it is not under a copyright more restrictive than CC-BY, please include a copy, in both the original language and English, as Supporting Information, or include a citation if it has been published previously.

**Comments of the authors:** We have provided more information about the scale and we will be sending the scale in the support information, in its original version (Spanish) and in the English language.

3. **Comments of the reviewers:** In the Methods, please discuss whether and how the questionnaire was validated and/or pre-tested. If these did not occur, please provide the rationale for not doing so.

**Comments of the authors:** We have clarified this item in the methods section.

4. **Comments of the reviewers:** We suggest you thoroughly copyedit your manuscript for language usage, spelling, and grammar. If you do not know anyone who can help you do this, you may wish to consider employing a professional scientific editing service.

**Comments of the authors:** We have improved it.

5. **Comments of the reviewers:** the introduction on the proposed generalized linear model is somewhat vague.The authors are suggested to explicitly specify the formulations of the proposed methods and illustrate why they are suitable to model the use of seat belts.

**Comments of the authors:** The generalized linear regression model was performed, using the Poisson family with robust variance to find the risk factors associated with greater or lesser use of a seat belt (outcome / main / dependent variable). This model was preferred due to the study design (cross-sectional), due to the dichotomous outcome, and to unify the statistical models, relating the random variable (use of a seat belt) with the systematic component (other independent variables evaluated), thus adjusting the model by potential confounding variables. This generalized linear model is proposed by the Peruvian transport authorities to make estimates of accidents and other issues related to road safety in transport, [1] as well as studies that evaluate behaviors dependent on drivers. [2]

Our study does not directly assess the characteristics of traffic accidents, nor the trend of these, however, the use of seat belts is part of the road safety devices that can have a decrease in the rates of morbidity and mortality in the drivers.

6. **Comments of the reviewers:** Some references on univariate/bivariate/multivariate generalized linear models should be added.

**Comments of the authors:** We have added the suggested references.

7. **Comments of the reviewers:** More discussion about the methods used in this field is needed. And more references about different methods could be added.

**Comments of the authors:** We have added the suggested references.

8. **Comments of the reviewers:** A table of the basic statistical information of the variables are needed.

**Comments of the authors:** We have added the basic statistical information of the variables evaluated in public transport drivers in table 01

9. **Comments of the reviewers:** The English writting could be improved.

**Comments of the authors:** We have improved it.

**References**

1. Ministerio de Transportes y Comunicaciones. Manual de Seguridad Vial. Lima - Perú; 2017. Avaible in: <https://portal.mtc.gob.pe/transportes/caminos/normas_carreteras/documentos/manuales/Manual_de_Seguridad_Vial_2017.pdf?fbclid=IwAR37pfT4iC6gUom_o-0ceucgVt2qwlrX5ICH7YgvwSvEIEjAMLfjimyo1P8> (accesed on 30/03/21)
2. Gershon P, Ehsani J, Zhu C, O'Brien F, Klauer S, Dingus T, et al. Vehicle ownership and other predictors of teenagers risky driving behavior: Evidence from a naturalistic driving study. Accid Anal Prev. 2018;118: 96-101.
